# Modern Technologies for Creating Synthetic Antibodies for Clinical application

**Published:** 2009-04

**Authors:** S. M. Deyev, E. N. Lebedenko

**Affiliations:** 1 Shemyakin and Ovchinnikov Institute of Bioorganic Chemistry, Russian Academy of Sciences, Moscow

## Abstract

The modular structure and versatility of antibodies enables one to modify natural immunoglobulins in different ways for various clinical applications. Rational design and molecular engineering make it possible to directionally modify the molecular size, affinity, specificity, and immunogenicity and effector functions of an antibody, as well as to combine them with other functional agents. This review focuses on up-to-date methods of antibody engineering for diagnosing and treating various diseases, particularly on new technologies meant to refine the effector functions of therapeutic antibodies.

## INTRODUCTION

A century ago, antibodies were regarded a "magic bullet" that would selectively strike disease hotbeds in the human organism (see Paul Ehrlich's Nobel Lecture of 1908 [1]). However, Paul Ehrlich's idea was put into practice only in 1975, when Köhler and Milstein's study initiated the development of monoclonal antibody technology. This technology makes it possible to produce not just a set of diverse immunoglobulin molecules (natural antibodies), but also a monospecific antibody focused on one specific antigen (monoclonal antibody, Mab) in response to antigen-driven immunization. This method is still the cornerstone of antibody reshaping. Unfortunately, the first attempts to use mouse Mab for clinical purposes were not successful and revealed the following, virtually insurmountable disadvantages of Mab: in some cases, its antibody affinity is lower than that of polyclonal antiserum; it has a high immunogenicity to humans and, as a consequence, is rapidly eliminated from the body; and it is unable to activate the complement system and cellular mechanisms of the immune response in a foreign environment. Nevertheless, after three decades of battles and defeats, hopes and PR blitzes, Mab proved to be a successful medicinal product from both the clinical and commercial standpoints (Table 1). The unique potential of immunoglobulins characterized by modular structures and functions related to other structural modules was realized, and the antibodies were modified for variable clinical applications thanks to the technologies of genetic engineering and transgenic animals. Depending on the practical task, researchers can directionally modify the molecular size, specificity, affinity, and valency; they can decrease immunogenicity and refine pharmacokinetic properties and effector functions. Moreover, antibodies are obtained as recombinant-fused proteins which include other specific antibodies, cytokines, protein toxins, radioisotopes, ferments, and fluorescent proteins. Currently, about 30 antibody medicines are approved for clinical application, 89% of which are used in treating oncological and immunological diseases. antibodies are also used in treating cardiovascular, autoimmune, and infectious diseases (Table 2). On the pharmaceutical market, antibodies come in second after vaccines in production volume. By 2011, the sales volume of antibody medicines is predicted to increase to $21 billion (Table 1). More than 85% of antibodies approved for clinical application are products of antibody reshaping. The approved antibodies include chimeric, humanized, and human Mab; antibodies obtained using phage display; and genetically engineered antibody conjugates with cytokines and toxins. Hundreds of antibody derivatives are still subject to clinical testing, including synthetic antibodies produced by gene engineering: bispecific antibodies; single-chain full-sized antibodies; different variants of truncated antibodies, including dimers and monomers of Fab fragments, scFv-fragments (single-chain mini-antibodies), single-domain antibodies (nanoantibodies), etc. Different technologies that make it possible to modify immunoglobulin molecules for certain clinical purposes are considered. This review is focused on antibody reshaping for the treatment and detection of oncological diseases, because this sphere is in particular need of these medicines. 

**Table 1:** Commercial success of several MAb used in oncology [Deonarain, 2008].

Commercial/USAN1 antibody name	Sales in 2005Р2006, US$, mln	Increase in sales relative to previous year, %	Evaluation of sales market in 2011, US$, mln
Rituxan©/rituximab	3800	16	6300
Herceptin©/trastuzumab	3100	82	4800
Avastin©/bevacizumab	2400	77	7800
Erbitux©/cetuximab	1100	57	2100
1 Currently, the nomenclature of monoclonal antibodies and their fragments approved in the USA is used around the world (United States Adopted Names (USAN); www.ama-assn.org), see Table 2.			

## 1. THE STRUCTURE OF A NATURAL ANTIBODY AND WHY IT NEEDS MODIFICATION FOR CLINICAL PURPOSES.

**Fig. 1.**
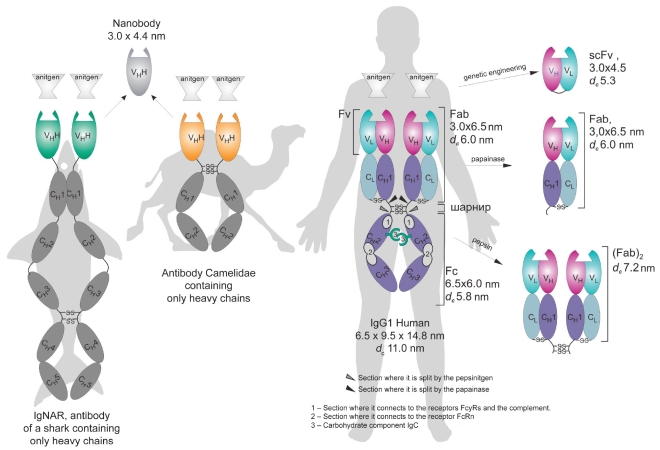
Natural antibodies and their fragments. Fab and (Fab)2 are antigen-binding IgG fragments produced by papain and pepsin hydrolysis, respectively; Fc is the C-end part of IgG composed of CH2 and CH3 constant domains of heavy chains responsible for effector functions; Fv is the variable fragment composed of variable domains of light (VL) and heavy (VH) chains; scFv is the single-chain variable fragment composed of VL and VH (conjugated by the gene engineering method); VHH is the nanoantibody, variable domain of cartilaginous fish and Camelidae antibodies containing only heavy chains; -S-S- is the disulfide bond. The indicated linear dimensions of antibodies and their fragments were measured by force microscopy methods [2, 3]; hydrodynamic diameter dc was calculated by the Stokes-Einstein formula [4, 5].

Antibodies, also known as immunoglobulins (IgG), are high-avid molecules which can detect and eliminate foreign antigens from the human organism. Most mammal antibodies have similar structures and are bivalent multidomain proteins with two antigen-binding sites (see, for example, human G immunoglobulin (IgG), Fig. 1). This compound Y-shaped molecular complex consists of two identical heavy (~50 kDa) and two identical light (~25 kDa) chains. Four inter-domain disulfide bonds provide stable conditions for the whole molecular complex. Globular structural immunoglobulin domains with a characteristic β-folded structure are stabilized by intradomain disulfide bonds and are subdivided into variable (V) and constant (C) domains. N-tail domains of light and heavy chains (VL and VH variable domains) vary significantly for different antibodies, while the remaining part of the polypeptide chains (CL and CH constant domains) varies for different antibody classes (and within one antibody class, depending on the species). The structural domains of immunoglobulin are isolated in space and perform different functions in the immune response process. 

Each antibody is characterized by a private specificity and a high affinity (KD 10^-8^-10^-11^ M) to its antigen, which is provided by the complementarity of the antigen-binding site to the definite antigen molecule site (epitope). Each antigen-binding site is composed of two variable domains - VH and VL - which are referred to as heavy and light chains of immunoglobulin, respectively. Each variable domain contains three complementarity determined regions (CDR). The ability of antibodies to bind two antigens simultaneously significantly increases the functional affinity (avidity) of immunoglobulins and the retention time on the surface cell receptors and other polyvalent antigens. In all, humans are known to have five classes of antibodies (IgM, IgG, IgE, IgD, and Iga). Not long ago, some animals (camel, llama, and shark) were revealed to have nonclassic antibodies without any light chains and with two antigen-binding sites, each of which is made up of only one heavy chain variable domain. The camel's variable domains were called "nanobodies" (Fig. 1).

The constant part of immunoglobulin consists of one light chain (CL) domain and three or four (depending on the class) heavy chain (CH) domains. Hydrophobic sites on the boundary of CH1 and CH2 domains remain mobile and form a link region, which provides the displacement and rotation of Fb-fragments. The heavy chain constant domains are, as a rule, glycosylated. The type of glycosylation varies significantly for different species and influences the immunoglobulin effector functions. Moreover, heterogeneous glycosylation is characteristic for the antibodies of one isotype within one species.

The IgG constant domain contains binding sites with protein of the C1q complement system and Fc-fragment cell receptors (FcyR), which mediate the effector (secondary) functions of immunoglobulins, i.e., their ability to kill target cells, activating the mechanisms of antibody-dependent cellular cytotoxicity (ADCC) and complement-dependent cytotoxicity (CDC). The ability of IgG and IgM to bind to neonatal Fc-receptor (FcRn) plays a very important role in controlling the antibody level in the blood serum. Hence, in the immune response process, an organism produces a complex set of heterogeneous molecules, which can include several cell mechanisms and a series of reactions for eliminating foreign antigens. 

Modern antibody reshaping is based on the monoclonal antibody technology put forth by Köhler and Milstein in 1975 [7]. This method involves the fusion (hybridization) of immune lymphocytes (splenicytes) which synthesize antibodies of the required specificity with the immortal myeloma cell line. The cell line obtained (hybridome) secretes monoclonal antibodies (Mab), i.e., antibodies of the same species, specific to the antigen used for immunization. The in vitro cultivation of hybridome or the propagation similarly to ascite in a mouse makes it possible to have constant sources of antibodies with definite specificities. The fusion of hybridomes characterized by various specificities enables one to obtain so-called quadroms, which produce - along with the primary Mab-monoclonal bispecific antibodies capable of binding with two different antigens. 

The introduction of monoclonal antibody technology led to great progress in using antibodies for both research and practical purposes (in vitro diagnostics). However, the hopes for creating specific and highly efficient anticancer drugs on the basis of Mab and their conjugates with active agents and for the rapid replacement of serum-derived vaccines with the Mab medicines, which would eliminate pathogenic microorganisms and toxins, were snuffed out. The idea of using Mab for the delivery of radioactive isotopes to target cells proved imperfect as well. It emerged that Mab have some properties which make them ineffective and dangerous for clinical use. at the first stages, individual Mab produced from a hybridome pool were often specified by a less efficient interaction with antigen than polyclonal antiserum dilution. The reason for this is the interaction between individual Mab and the sole antigenic epitope and, probably, the partial inactivation of antibodies in the release process. Full-size Mab have non-optimal pharmacokinetic properties for application as targeting molecules. as foreign proteins, mouse Mab are subject to rapid catabolism. Moreover, they have almost no interaction with the FcRn receptor, which is responsible for the recirculation of immunoglobulins from lysosomes, which speeds up the Mab removal from the blood flow. On the contrary, full-size Mab circulate in the blood flow for up to 2 weeks. Due to their high molecular weight (150 kDa), immunoglobulins are slowly distributed in the organism, have poor penetration ability, and are not cleansed from the body through the kidneys. The xenogenic nature of Mab impedes the activation of a complement system and the cell mechanisms of foreign antigen elimination (ADCC and CDC) due to the poor identification of the mouse Mab by Fc-receptors of human immune system cells. The high immunogenicity of mouse Mab is dangerous for a human being and cannot be used for clinical purposes due to the risk of hyperimmune reaction formation. Serious side effects related to the nature of the toxin attached were also noted when the first generation of immunotoxins was used. It ought to be noted that the Mab medicines successfully used in medical treatment today have dangerous side effects (Table 2).

**Table 2:** MAb medicines approved for clinical use and possible side effects.

Application field	Commercial name	USAN nomenclature name^2^	Antibody format	Target	Application, action mechanism (↑ in-crease or ↓ decrease in effect)	Company and registration year	Possible side effects [http://www.i-sis.org.uk/WOFAMAD.php]
Therapy of tumoral diseases	Avastin©	Bevacizumab	Humanized IgG1	VEGF	Intestine cancer. Binding with ligand, antagonist. Angiogenesis↓, metastasis↓.	Genentech, 2004	Gastro-intestinal perforations and wound disruption, occasionally with a lethal outcome.
	Bexxar©	^131^I-Tositumo-mab	Mouse ^131^I-IgG2a	CD20 (B-cells)	Non-Hodgkin lymphoma. Radioimmunotherapy, ADCC, CDC.	GlaxoSmith-kline, 2003	Hypersensitivity reactions, including ana-phylaxis.
	Campath©	Alemtuzumab	Humanized IgG1	CD52	B-cell chronic lymphocyte leukemia.	Genzym/Schering, 2001	Decrease of blood-forming functions of bone marrow, occasionally serious to lethal outcome.
	Erbitux©	Cetuximab	Chimeric IgG1	EGFR	Metastatic cancer of intestine, head, and neck.
	Receptor antagonist. Apoptosis↑, chemo- and radiosensi-tivity↑, prolif-eration↓, angiogenesis↓, metastasis↓.	Im-clone/Bristol-Myers Squib, 2004	Anaphylactic reactions (3% of cases) (bronchial spasm, hoarse breath, hy-potension), rarely lethal outcome (1 case in 1,000).
	Her-ceptin©	Trastuzu-mab	IgG1	HER2	HER2-positive metastatic breast cancer. Prolif-eration↓, angiogenesis↓, chemosensitivity↑.	Genentech, 1997	Cardiomyopathy.
	Mylotarg©	Gentuzumab ozogamicin	Conjugate of human-ized IgG4-calicheamicin	CD33	CD33-positive acute myeloid leukemia.
	Cell intoxica-tion due to induction of DNA breaks.	Wyeth pharma-ceuticals, 2000	Heavy reactions of hy-persensitivity, including anaphy-laxis, hepatoxic-ity, and hema-tologic toxicity.
	Prostas-cint©	Capromaab pentetate	Mouse ^111^In-IgG1	PSMA, prostate specific membrane antigen	Diagnostics of prostate can-cer	Cytogen, 1996	Anaphylactic or anaphylactoid shocks at single dosing. Repeated dosing can cause danger to life due to serious systematic reactions of cardiovascular, respiratory, and nervous systems (shock, heart and respiratory ar-rest, paralysis).
	Rituxan©	Rituximab	Chimeric IgG1	CD20 (B-cells)	Non-Hodgkin lymphoma. Chemosensitivity, ADCC, CDC	Genen-tech/Biogen, 1997	Reaction to intravenous injection. Ana-phylactic shock with lethal out-come.
Vectibix©	Panitumu-mab	Human IgG2	EGFR (HER1)	Intestine can-cer. Receptor antagonist. Apoptosis↑, chemo- and radiosensi-tivity↑, prolif-eration↓, angiogenesis↓, metastasis↓.	Amgen, 2006	No data
Zevalin©	Ibritumo-mab tiuxetan	Mouse ^90^Y-IgG1	CD20 (B-cells)	Non-Hodgkin lymphoma.Radioimmunotherapy, apop-tosis↑	Biogen IDEC, 2002	Scheme of treatment with the medicines in-cludes Rituxan© (see above).
Transplantology	Orthoc-lone OKT3©	Muromonab-CD3	Mouse IgG2a	CD3 (T-lympho-cytes)	Prevention of acute rejec-tion at renal trans-plantation. Blocking of T-killers activity.	Ortho Biootech, 1986	Anaphylactic or anaphylactoid shocks, includ-ing: cardiovascular collapse, heart and respiratory arrest, convulsions, brain edema, blindness, and paralysis.
	Simulect©	Basilixi-mab	Chimeric IgG1	CD25	Prevention of acute rejec-tion at renal trans-plantation. Blocking of IL-2R.	Novartis, 1998	Heavy anaphylactic re-actions (in 24 hours), including: hypotension, bronchial spasm, heart and res-piratory arrest, pulmonary edema, fever, and loss of consciousness.
	Zenapax©	Daclizumab	Humanized IgG1	CD25	Prevention of acute rejec-tion at renal trans-plantation. Blocking of IL-2R.	Roche, 1999	Heavy anaphylactic re-actions (in 24 hours), including: hypotension, bronchial spasm, heart and res-piratory arrest, arrhythmia, pulmonary edema, fever, and loss of consciousness.
Cardiovascular dis-eases	ReoPro©	Abciximab	F(ab)-chimeric fragment of IgG1	Complex of IIB-IIIA glycopro-teins	Thromboprophy-laxis. Antago-nist of IIB-IIIA glycopro-teins.	Centocor/Elli Lilly, 1994	Indigestion, burning sensation, belching, pruritus, numbness, nervous system disorder, mental debility.
	Monafram®		F(ab’)_2_ fragment of mouse IgG1	Complex of IIB-IIIA glycopro-teins thrombo-cytes	Thromboprophy-laxis at coro-nary angio-plasty.Antagonist of IIB-IIIA glycoproteins.	Framon Ltd, Russia, 2002	Bleeding, allergic reactions, thrombocytopenia (few cases of deep thrombocyto-penia), immune response in 5Р6% of cases
Therapy of autoimmune diseases	Humira©	Adalimumab	Human IgG1	TNFα	Rheumatoid ar-thritis, pso-riasis. Blocking of TNFα activity, inflammation↓.	Abbottt Labo-ratories, 2002	Tuberculosis, fungal invasion and other in-fections, acute myeloid leukemia.
	Raptiva©	Efalizumab	Humanized IgG1	CD11a	Psoriasis. Blocking of T-lymphocytes activity.	Genen-tech/Xoma, 2003	Hemolytic anemia, serious infections including tuberculosis pneumonia and bacterial sepsis at antimicrobic therapy
	Remicade©	Infliximab	Chimeric IgG1	TNFα	Crohn’s dis-ease, rheuma-toid ar-thritis, inflammatory diseases. Blocking of TNFα activity, inflammation↓.	Centocor, 1998	Tuberculosis infection, occasionally with lethal outcome.
	Tysabri©, Antegren©	Natalizu-mab	Humanized IgG4	α4 subunit of α4β1 and α4β7 integrins	Multiple scle-rosis. Inhibition of immune cells migration to inflammatory tissue.	Biogen Idec/Elan, 2004	Sales were stopped in 2005. Progressive leu-koencephalopathy in 1 case of extended medica-tion use.
	Xolair©	Omalizumab	Humanized IgG1	IgE	Allergic asthma.Inhibits egress of IgE from mast cells.	Genentech, 2002	Appearance or re-cidive of cancer.
Others	Lucentis©	Ranibizu-mab	Humanized F(ab) fragment of IgG1	VEGF	Age-related retina degen-eration.Blocking of angiogenesis.	Genentech/Novartis, 2006	Allergic reactions, intraocular inflammation, retinal hemor-rhage.
	Synagis©	Palivizu-mab	Humanized IgG1	F-protein of respi-ratory syncytial virus	Infection pre-vention with respiratory syncytial vi-rus.	Medimmune, 1998	Rarely acute hypersensitivity; repeated in-jection can cause (1 case in 100 thousand) anaphy-laxis.
^1^ Currently, the nomenclature of monoclonal antibodies and their fragments approved in the USA is used around the world (United States Adopted Names (USAN); www.ama-assn.org), see Table 2.								^2^ Currently, nomenclature of monoclonal antibodies and their fragments approved in the USA is used in the whole world (United States Adopted Names (USAN); www.ama-assn.org), see Table 2.							

The experience and new knowledge about the mechanism of interaction between antibodies and target molecules were gained through trial and error. It appears that some clinical cases require Mab modifications, often mutually antithetical. The technologies of gene engineering and transgenic animals, along with monoclonal antibody technology, made the greatest contribution to antibody reshaping. Let's provide some insight into the major directions of antibody engineering for clinical use. Currently, the antibody medicines approved by the Unites States system (US Adopted Names, www.ama-assn.org) are used (Table 3). 

**Table 3:** USAN nomenclature of therapeutic medicines with antibodies and their fragments (United States Adopted Names; http://www.ama-assn.org/"www.ama-assn.org).

Prefix	Disease or target	Antibody origin	Suffix	Examples
Appro-priate syllable(s) different from those used earlier	**vir** - virus **bac** - bacterial **lim** - immune system **les** - infection **cir** - cardiovascular **fung** - fungal **ner** - nervous system **kin** - interleukin **mul** - skeletomuscular system **os** - bones **toxa** - toxins **anibi** - angiogenesis Tumors: **col** - large guts **mel** - melanoma **mar** - lacteal gland **got** - spermary **gov** - ovary **pro** - prostate gland **tum** - different	**u** - human being **o** - mouse **a** - rat **e** - hamster **i** - primate **axo** - rat/mouse **xi** - chimera **zu** - humanized **xizu** - combination of chimeric and humanized chains	mab	
tras	tu	zu	mab	**Trastuzumab** (Herceptin©) - anti-tumor humanized MAb
ab	ci	xi	mab	**Abciximab** (ReoPro©) - chimeric MAb for thromboprophylaxis
r	anibi	zu	mab	**Ranibizumab** (Lucentis©) - humanized MAb to block angiogenesis
To create new designation, it is essential: To choose the appropriate syllable(s) different from any used earlier and to put it (them) at the beginning of a word; To add syllables in the following order: disease class (tumor type) or target, source of origin, suffix "mab"; To simplify pronunciation, the last consonant in the syllable designating the target may be omitted.				

## 2. TRUNCATED ANTIBODY FRAGMENTS AND ALTERNATIVE FRAMEWORK PROTEINS WITH SPECIFIC ANTIGEN BINDING

Originally, the most attractive property of antibodies in terms of the "magic bullet" construction was their specific binding with the antigen, which was assumed to allow the delivery of an active agent (for instance, a radioactive isotope) to a target molecule. The governing factors which would provide the efficiency of this delivery are the affinity and specificity of the antibody targeted to the antigen, as well as its physicochemical properties, such as valency, surface charge, and size [8, 9]. 

The specificity of an antibody constructed for therapeutic use depends on selecting the appropriate target molecule. The Mab approved for clinical use are directed against 15 targets, most of which are surface cellular antigens, including such widespread cancer markers as HER1, HER2/neu, and PSMa (prostate specific membrane antigen) (Table 2). The last decade was characterized by great progress in understanding the mechanism of antibody action and in developing new target molecules for treating oncological, autoimmune, infection, and cardiovascular deseases. 

Five groups of potential target molecules for antibodies in cancer treatment have been pointed out in recent reviews: cancer cells inside solid tumors, which are less treatable; diffusive cancerous cells (leukemia); the stroma associated with the tumor (fibroblasts); and blood vessels associated with the tumor and vascular endothelial growth factors (VEGF) [10, 11]. additional target molecules can appear in response to blocking surface receptors [12] or inducing cancer cells under the action of a medical agent [13]. 

The affinity of antibodies used in therapy today is within the nanomolecular concentrations (from 10^-8^ to 10^-10^ M), which are optimal for solving most problems. Increasing the antibody affinity to 10^-11^ M significantly decreases both its ability to penetrate the tumor and its targeting selectivity [14]. 

The size of the therapeutic antibody molecules is an important factor, because it determines the possibility and rate of renal excretion. The time of the renal half-excretion of protein correlates with the molecular size: the limit of glomerular filtration is estimated at 60-65 kDa [15]. The full-size IgG molecules (~150 kDa) (Fig. 1) are too large and cannot be excreted through the kidneys. In contrast, the scFv-fragments (~30 kDa) (Fig. 1) are characterized by 0.5-2 hours of half-removal period and readily leave the organism [16]. The monomeric Fab-fragments (48 kDa) hold an intermediate position, with 14-15.5 hours of half-removal time [16, 17]. 

The optimal surface charge for therapeutic antibodies is the interval of their isoelectric points from 5 to 9. an increase in the positive or negative charge complicates the antibody binding with the target cells [18]. 

The valency of the recombinant antibody targeted on the antigen plays a very important role in retaining Mab in the target cells. Most natural mammal antibodies are bivalent. The ability to simultaneously bind two antigens significantly increases the functional affinity, or avidity, of immunoglobulins and provides the long-term retention of antibodies on the cell surface receptors or polyvalent antigens. This property is used in the wild: tetra- and decavalent complexes of Iga and IgM are characterized by a higher affinity to the multipoint binding of polyvalent antibodies and can protect against natural polyvalent antigens, such as pathogenic microorganisms, more efficiently. 

Hence, using antibodies as targeting components for the delivery of diagnostic and therapeutic agents of, primarily, radioactive isotopes and as specific blockers of pathogenic processes required the elimination of their effector functions and a cardinal modification of their physicochemical properties. 

The first truncated fragments of antibodies-monomeric Fab and dimeric (Fab)2 (Fig. 1)-meant for use in radioimmunotherapy were produced by processing intact IgG with such proteolytic enzymes as papain and pepsin (Fig. 1). These fragments are absent in the Fc domain, which mediates the IgG effector properties that are undesirable in the current situation. Moreover, the considered fragments are not capable of recirculating from lysosomes, which causes the dose-limiting myelotoxicity and nonspecific radioactive background to decrease in comparison with the full-size IgG. However, the fragments obtained retain antigen-binding properties that are comparable with those of the parent antibodies. Examples of the therapeutic use of the monomeric Fab-fragment include ReoPro© antiaggregant (Abciximab) and its Russian analogue, Monafram© [19], which are applied in cardiosurgery for thrombosis prevention, as well as an angiogenesis blocker - Lucentis© (Ranibizumab) - used for treating age-related retina degeneration (Table 2). Because of its diminished size, Lucentis© (Ranibizumab) readily penetrates into the inner eye cover, unlike full-size antibodies, making it ideal for clinical application [17]. The main disadvantage of the enzymatic method of antibody fragment production is the complicated purification of the target product. Moreover, further modification of the antibody's physicochemical properties is possible only by chemical methods. Furthermore, the first generation of Fab-fragments was of mouse origin and characterized by high immunogenicity. 

Developing genetic engineering methods made it possible to simplify the preparation of truncated antibody derivatives. Recombinant antibodies, so called single-chain variable fragments (scFv) (Fig. 1), are coded by one gene and contain only one antigen-binding site, which consists of variable domains of light (VL) and heavy (VH) chains connected by an elastic peptide linker [20] (Fig. 1). These scFv-fragments are monovalent and are often specified by a lower affinity than the primary one. Their rapid removal from the organism and homogenic focus on the target makes them more appropriate for delivering radioactive isotopes than the parent full-size IgG. Other very important advantages of single-chain antibodies are as follows: their compatibility with bacterial expression systems, their reduced immunogenicity, and their absence of effector functions. Nevertheless, the monovalent scFv-fragments never gained universal currency in medical practice, because even a high-affinity but a monovalent binding with the antigen on the cell's surface does not provide long retention and leads to the rapid dissociation of antibody. 

Cloning single-chain antibodies retaining antigen-binding functions had significant consequences. From then on, the technologies for creating synthetic antibodies of almost any specificity were quickly climbing to new heights. Diverse collections (libraries) of recombinant antibody fragments were designed, new methods which could increase their diversity were offered, and technologies which made it possible to increase the affinity of antibodies and efficiently select new variants were developed [21, 22]. The selection platforms for immunoglobulin libraries were developed on the basis of phage, ferment, and ribosomal displays [23]. Nonclassical antibodies of cartilaginous fish and animals of the Camelidae genus (camel and llama), containing only heavy chains, were the perspective source for antigen-binding fragments with uncommon properties (Fig. 1). an antigen-binding site of such antibodies was formed by the sole variable domain, which is characterized by good solubility, high stability, and the ability to bind with difficult-to-access antigens [24, 25].

That period was also marked by the intensive development of molecular constructions-alternatives to the binding domains of antibodies - which were called scaffolds in English-language scientific literature. This term was offered by a. Plyuktun [26] for designating a protein carcass or framework bearing altered amino-acid residues or small sequences, which provided different protein variants with various functions, commonly, the possibility of effective binding with specific target molecules. The word scaffold is translated into Russian as a construction for capital punishment or as construction trestles. "Construction trestles" is the implied meaning, but it is hardly possible to use this phrase in the context of protein engineering. Therefore, we offer the terms framework protein or framework peptide along with the English calque scaffold.

Typical natural framework proteins or scaffolds are antibodies or T-cell receptors. There are many other natural and synthetic framework proteins, for instance, affibody, peptabody, ankyrin repeats, and lipokalins [26]. Developing alternative framework compounds is a perspective direction in research for creating new compounds to selectively bind to target molecules and cells. New compounds are meant for biomedical use and are appropriate for robotic technologies and the construction of supramolecular nanostructures. These facts are confirmed by the creation of the ProteomeBinders association in Europe, which is focused on thoroughly studying human proteome using high-affinity reagents [27]. 

**Fig. 2.**
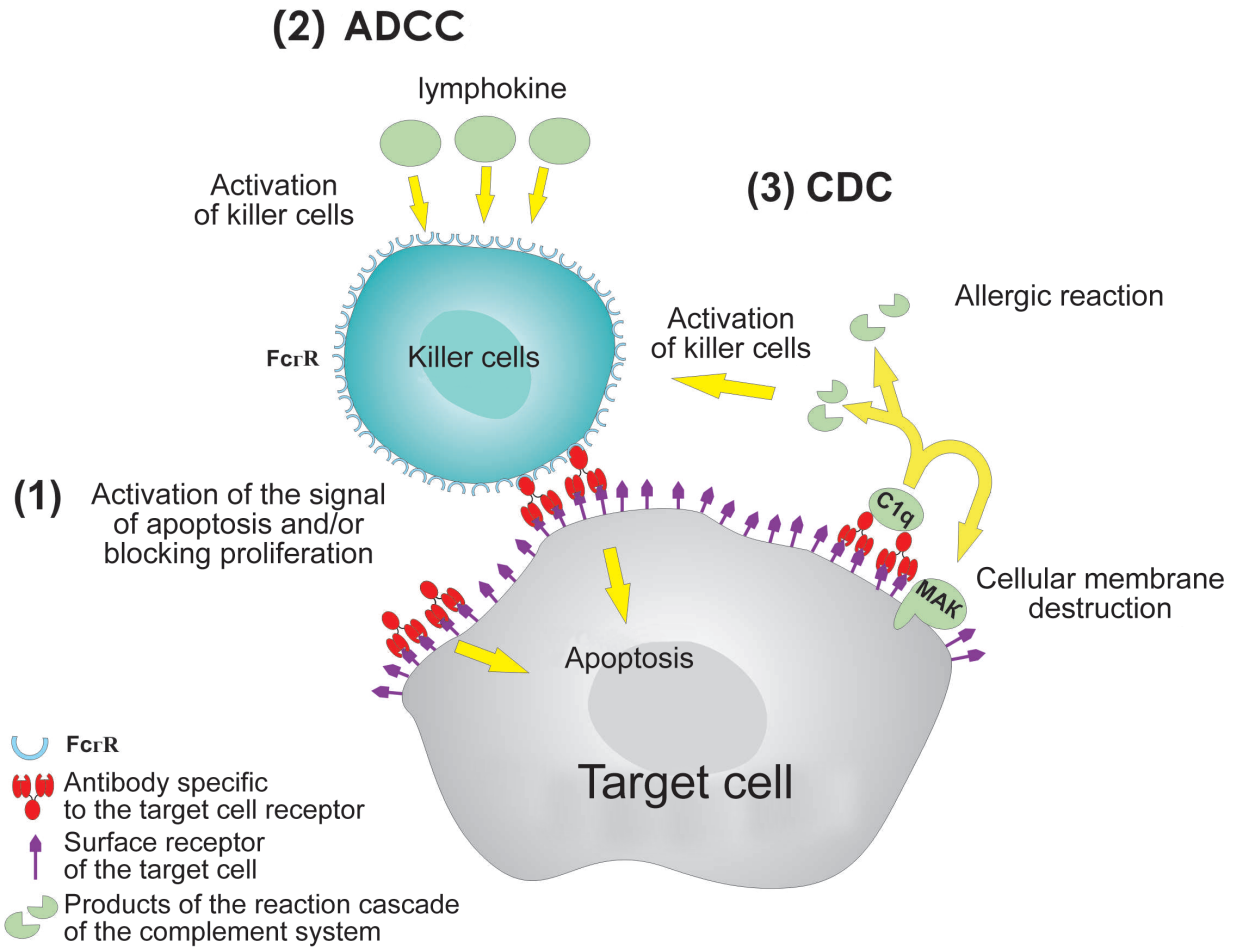
Interaction Pattern of Unloaded antibodies and Target Cell. (1) antibodies can cause apoptosis or block the proliferation of target cells, binding with membrane antigens on their surface (membrane raft mechanism) [6]. (2) antibody-dependent cellular cytotoxicity (ADCC). Killer cells carrying receptors of IgG FcyRI (CD16), FcyRII (CD32), FcyIII (CD64) constant domains (natural killers, killers activated by lymphokines, macrophages, and phagocytes) and receptors of IgE FcεRI and FcεRII (CD23) constant domains (acidocytes) on their surface attack the target cell, whose surface antigens were bound with antibodies. (3) Complement-dependent cytotoxicity (CDC). antibodies conjugated in pairs bind to protein C1q complex, causing a cascade of reactions of the complement system, which leads to membrane destruction. Some products of this reaction cascade involve immune cells or cause allergic shock.

## 3. GENE ENGINEERING TECHNOLOGIES FOR OPTIMIZING BIODISTRIBUTION AND PHARMACOKINETICS.

a wide range of theoretical and experimental data testifies to the fact that the simplest method to overcome the imperfection of the recombinant single-chain Fv-fragments is a return to multivalency, which is a primary characteristic property of antibodies. Indeed, every effort was spent to construct multimeric formats of truncated antibodies in order to optimize their pharmacokinetic properties and to improve the biodistribution of the active agents delivered by antibodies. Currently, a wide variety of such constructions is subject to clinical testing [28, 29]. 

In the past decade, a wide range of methods was developed in engineering multivalent antibodies; they are described in detail in our review [9]. The valency of the constructed multivalent derivatives, i.e., the number of antigen-binding units, may vary from 2-10. The offered strategies are based on the chemical conjugation of antibody fragments, the use of self-associating peptides, and the phenomenon of domain shuffling when two antibodies of different specificities are mixed. antibodies, both bivalent tandem and bispecific are obtained by linear gene fusion. Using heterodimerization modules, including the streptavidin-biotin system, the barnase-barstar module [30, 31], and the "knob-and-holes" [32] and "dock and lock" [33, 34] methods, is more universal. Each strategy has advantages and disadvantages, but none of them is universal. The systematization and comparative analysis of the experimental data on the pharmacokinetics of multivalent antibody derivatives of different formats focused on HER2/neu and CEa cancer markers and on angiogenesis markers, such as the ED-B domain of fibronectin, showed that pharmacokinetic characteristics and biodistribution are significantly improved when monovalent antibodies are replaced with bivalent (multivalent) varieties [9]. 

One more important method of varying the Mab pharmacokinetic characteristics is a delicate site-specific modification of the constant domain depending on certain applications of the immunoglobulin constructed. Neonatal or so-called "salvage" Fc-receptors (FcRn) perform the following important functions in an organism: they transport IgG through epithelial and endothelial barriers, they transmit immunoglobulins from mother to child, and they protect IgG and albumin against catabolism in lysosomes of vascular endothelial [35]. FcRn is characterized by a high affinity to IgG at pH 6.0 and does not bind with it at pH 7.2. Hence, the mechanism of IgG prevention against catabolism is based on the binding of FcRn with antibodies in lysosomes at pH 6.0 and returning to the blood flow [36]. Directed mutagenesis of the Fc-fragment makes it possible to produce different variants of antibodies, randomly changing the time of their half-life in the blood flow [37]. Replacing key amino-acid residues in the constant domain sites of mouse anticancer antibodies that are involved in the interaction with FcRn allowed several mutants to be prepared with a half-life time from 8 to 80 hours in the mouse's organism. The same indicator is ~12 days for wildlife antibodies [38]. These antibodies are readily removed from the organism, which is of critical importance for radioimmuno therapy and improves the resolution when the scintigraphy methods are used. On the other hand, mutant antibodies with a higher affinity to binding with FcRn at pH 6.0 are specified by a longer half-life in the organism [39]. Increasing the circulation time is important when using therapeutic antibodies to treat infectious diseases or neutralize antibodies for treating acute intoxications. For instance, injecting triple mutation M252Y/S254T/T256E into the Mab constant domain specific to the respiratory syncytial virus increased their binding by ten times at pH6.0 with FcRn of a human being and a crab-eating macaque (Macaca fascicularis). at the same time, mutant Mab are readily separated from antibodies at pH 7.4. Investigating the pharmacokinetic properties of mutant Mab on the basis of a primate model showed that the lengths of both their half-life in the blood flow and accumulation in the lungs increased 4 times [40]. It ought to be noted that mutagenesis of the constant domain amino-acid residues responsible for binding with FcRn commonly does not influence other effector properties of antibodies. Hence, directed alteration of the FcRn-binding sites allows the control of the pharmacokinetic properties of Mab constructed for different purposes. Moreover, creating peptide antagonists, which block the FcRn receptor, may be done to decrease the level of pathogenic IgG in the organism, which is one more method of autoimmune disease treatment [41]. 

an alternative method to improve the pharmacokinetics and biodistribution of mini-antibody derivatives is adding polyethyleneglycol [42] and albumin [43], which would increase the molecular weight of the compound and, respectively, decrease renal excretion. It is quite possible that when albumin is added, additional recirculation mechanisms mediated by the neonatal receptor are activated [44]. 

## 4. THE REDUCTION IN THE IMMUNOGENICITY OF MONOCLONAL ANTIBODIES (HUMANIZATION).

One of the first problems arising in the course of creating antibodies for clinical purposes is the high immunogenicity of Mab, which is xenogenic for a human being. The human anti-mouse antibodies response (HaMa) causes serious systematic reactions in the human organism up to allergic shock (Table 2). Using gene engineering methods allowed the partial replacement of the mouse Mab immunogenic sites with the corresponding fragments of human antibodies. 

**Fig. 3.**
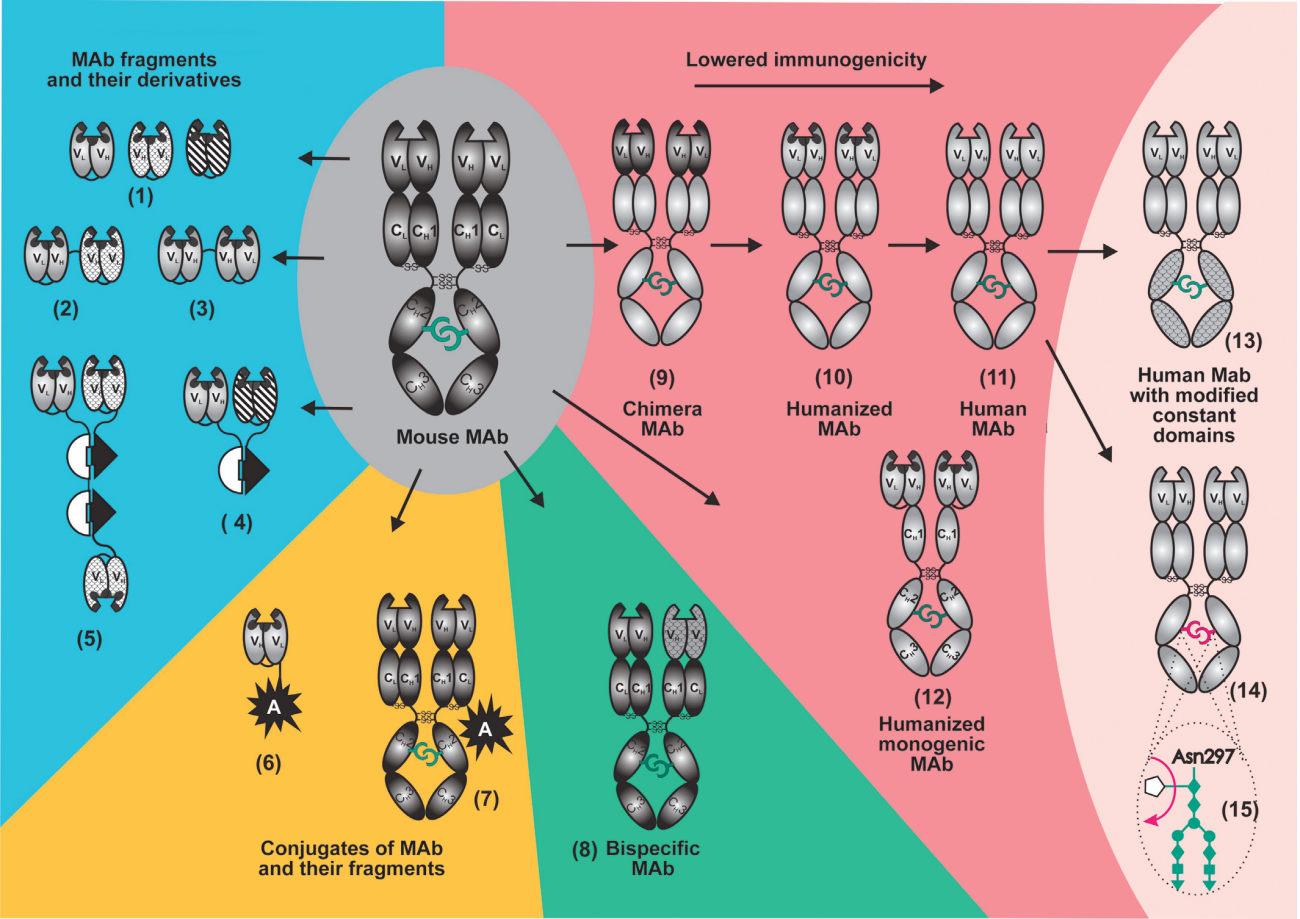
Modification of mouse monoclonal antibodies (Mab) for clinical application. The following Mab fragments produced by gene engineering are considered: (1) single chain antibodies (scFv or mini-antibodies) of different specificity and composed of VL- and VH-domains bound by peptide linker; (2) bispecific mini-antibody; (3) dimeric mini-antibody; (4, 5) dimmer and trimer of mini-antibody bound by the barnase-barstar module; (6) conjugate of mini-antibody with bioactive agent produced by the gene engineering method; (13) human Mab with modified constant domains; (14) human Mab with modified carbohydrate component (15). Legends of antibodies and their fragments are as in Fig. 1; hypervariable sites of variable domains (CDR) are indicated with black color; mouse antibodies and fragments, with dark gray; human antibodies and fragments, with light grey; variable domains of different specificity (1-5) and modified constant domains (13) are indicated with hatching. White semicircle and black triangle denote barnase and barstar, respectively. A denotes the bioactive component (radioactive isotope, toxin, ferment, fluorescent protein, etc.).

In chimeric Mab [45] (Fig. 3), all constant domains of mouse antibodies are replaced with constant domains of human immunoglobulin. In humanized Mab (Fig. 3), only hypervariable sites responsible for complementary interaction with antigen (CDR) are of mouse origin. Monogenic constructions of humanized antibodies are produced by gene engineering methods as well [46]. Currently, pure mouse antibodies are applied in small doses only in radioimmunotherapy, due to the necessity of rapid excretion from the blood flow (Bexxar© and Zevalin© medicines in Table 2). 

More complex technologies were developed to obtain pure human Mab (Fig. 3). Screening antibody scFv or Fab-fragments of the desired specificity from the combinatorial libraries (phage, ribosome, and ferment displays) with the following reconstruction of full-size human immunoglobulins from them does not require preliminary immunization. The adalimuMab (Humira©) medicine widely used in treating autoimmune diseases was initially obtained as a scFv-fragment by the Cambridge antibody Technology (MedImmune) and then restored to the formation of an antibody [47]. Pure human Mab are also created in transgenic mice with the expressing genes of human immunoglobulins. The technology involves replacing mouse IgG gene locuses with their corresponding human genome sites. One example is Panitumumab (Vectibix©), specific to EGFR, made by HuMouse technology (abgenix).

Currently, this sphere is being actively developed, and all the many methods used for antibody humanization may be conditionally subdivided into rational and empiric [48]. The rational methods involve a so-called design cycle: generate a few variants based on data on the structure of the antibody and its gene, followed by analyzing the variants, and then selecting the best variant. The rational methods include the following procedures: grafting hypervariable sites of the mouse Mab responsible for the complementarity of interaction (CDR-grafting) or only 20-30% of those responsible for specificity (SDR-grafting) into the framework of the human immunoglobulin [49]; resurfacing mouse Mab to make it similar to the human immunoglobulin surface; and superhumanization based on the detection and elimination of potential epitopes from the mouse immunoglobulin molecule for a major complex of histocompatibility and T-cells, i.e., human string content optimization [50]. Recently, the single-domain antibodies of a camel (nanoantibodies) (Fig. 1), which are characterized by high stability, technological effectiveness, and the ability to bind hard-to-access antigens, have drawn the attention of researchers. The general strategy of humanization was developed to apply those nanoantibodies for clinical purposes [51]. Researchers revealed amino-acid residues which determine the distinctions of nanoantibodies and the corresponding heavy chain variable domain of human IgG. Then, nanoantibodies taken from the representative library were humanized in the course of the sequential replacement of several sites in the molecule. after that procedure, nanoantibodies remained stable enough and kept their primary affinity. 

In contrast to the rational methods, empiric methods of humanization are based on the creation of large combinatorial libraries [21, 22] and selecting the required variants using such processing technologies as phage, ferment or ribosomal displays, or high throughput screening methods [23]. These methods may be applied mostly to single-chain antibodies (scFv), which, when needed, may be transformed into monogenic Mab [46] (Fig. 3, 8) by fusion with the Fc constant domain and dimerization or they may be reconstructed into a full-sized antibody with a classical structure. 

As a rule, both rational and empiric methods complement each other. Currently, about 40 humanized and human Mab obtained by new selection methods for cancer treatment are undergoing the last stages of clinical testing [47]. The humanization of therapeutic antibodies not only weakened the dangerous side effects, but also allowed more complete use of the powerful potential of Mab, i.e., the involvement of effector functions. 

## 5. THE OPTIMIZATION OF ANTIBODY EFFECTOR FUNCTIONS. 

Antibodies bound to the surface of the target cell can cause its death by triggering the ADCC and CDC mechanisms (see above) (Fig. 2) . Far from all clinical applications of Mab find these mechanisms useful. For instance, the Mab effector functions are not required for the directed delivery of radioimmunoisotopes or blocking transplant rejection; moreover, they can cause dangerous complications. In other cases, the action of Mab may be intensified with the aid of killer cells. One such method was creating bispecific Mab, which attracted cytotoxic T-lymphocytes to the target cells (see Section 6). Moreover, in recent years researchers have detected a wide range of immunocyte receptors responsible for the interaction with antibodies and determining the role of IgG sites in this interaction. Recent investigations have showed that the efficiency of a therapeutic antibody depends on its affinity to FcyRIIIa (CD16) and FcyRIIa (CD32), which is characteristic for the activating receptors of most killer cells. It became apparent that the reaction of a patient to treatment by Mab medicines depends on his phenotype. Patients with FcyRIIIa-158 V/V and FcyRIIa-131 H/H phenotypes are at an advantage when being treated for follicular and non-Hodgkin lymphoma with Rituxan© (Abciximab) [52]. analogous results were obtained when breast cancer was treated by Mab Herceptin© (Trastuzumab) [53]. On the other hand, a therapeutic antibody can bind to a FcR receptor on non-toxic cells (thrombocytes and B-cells), which are able to inhibit the activation of effector killer cells (for instance, the FcyRIIb receptor on macrophages). Experiments on animals show that the FcyRII inhibitor isoform decreases the therapeutic effect of humanized Herceptin© (Trastuzumab) [54]. The presented data testify to the fact that, at the current stage of gene engineering, it is essential to optimize the effector functions of the antibody depending on the sphere of application in order to increase the efficiency of the therapeutic treatment. 

It is also necessary to mention the constructions where a constant part of immunoglobulin is used in combination with targeting peptides (instead of the IGG antigen-binding sites) [55] or with monomeric and dimeric cytokines to improve pharmacokinetics and protect against degradation [56].

There are several methods to optimize the antibody's effector functions. To deactivate these functions, scientists use different variants of truncated antibodies depleted in the constant domains responsible for binding to the Clq component and killer cells (see Section 3). Examples of truncated antibodies are ReoPro© (Abciximab) and Monafram© antiaggregants, as well as the antigenesis blocker Lucentis© (Ranibizumab) (Table 2). another method is replacing IgG1 with IgG2 like in the cancer medicine Vectibix© (Panitumumab) specific to the EGFR marker (HER1) [57] (Table 2). The IgG2 constant domain almost doesn't bind to FcyR receptors of the killer cells at all: therefore, antibodies of this subclass cannot cause ADCC.

A more universal approach involves the directed mutagenesis of constant domain binding sites with C1q and FcyR cell receptors. Directed mutagenesis was made possible due to the accurate mapping of FcyR binding sites on the IgG1 constant domains [58]. Researchers managed to obtain a whole spectrum of mutant Mab from antibodies which barely bound to FcyR and C1q and are therefore not able to cause ADCC and CDC, up to high-affinity antibodies characterized by a whole range of reactions caused by those two mechanisms [59-61]. Researchers also created Mab variants that were not able to bind to C1q and cause reactions of the complement but could retain a high affinity of binding to FcyR receptors and the ability to cause ADCC [50]. The important result of that investigation was the creation of Mab (S239D/I332E/a330L) which could readily bind not only to the rare homozygotic allele of FcyRIII-158V/V (20% of patients), but also to more ubiquitous alleles, such as FcyRIII-158V/F (45% of patients) and FcyRIII-158F/F (35% of patients). The construction of therapeutic antibodies with effector functions independent of patients' polymorphism makes it possible to successfully treat all patients with medicines such as Herceptin© (Trastuzumab) and Rituxan© (Abciximab).

IgG contains only 3% of carbohydrate (Fig. 1); however, in spite of its insignificant content, this component plays a very important role in the performance of antibody effector functions. IgG contains two branched oligosaccharide chains, each of which is joined through a nitrogen atom to asn297 residue in the constant CH2-domain (Fig. 3). The carbohydrate composition is characterized by high species specificity and varies within one species. The glycosylation of synthetic (or constructed) antibodies depends on the system in which they were prepared, the selected clone, and the separation system. Therefore, the "incorrect" glycosylation of therapeutic antibodies obtained in xenogenic systems (see Section 8) can cause an immune response, allergic reactions, and favors the rapid removal of these antibodies from the human blood flow [62]. Oligosaccharides (glycans) are known to be the key components in the IgG composition, because they provide the optimal binding of IgG constant domains with the FcyR receptors mediating the effector functions. It has been proven recently that the removal of fucose from antibody carbohydrate increases the aCDC by over 50 times [63]. a decrease in the antibody efficiency in the organism (when compared to the in vitro experiments) forces the concentrations of the medicine injected to increase. This situation is related to the inhibitory effect of fucosylated immunoglobulins in the blood plasma [64]. Non-fucosylated therapeutic antibodies are characterized by a higher affinity to FcyR receptors than fucosylated ones, which allows them to withstand the inhibitory effect of IgG circulating in the blood plasma and to be effective at lower concentrations [65]. 

All therapeutic antibodies approved for clinical application are produced today in the CHO cells, in mouse myeloma cells such as NS0 and SP2/0, and in mouse hybridomes. almost all of them have fucosylated carbohydrate components and, as a result, non-optimal aCDC [66]. The fucosylation of the IgG carbohydrate component is carried out in mammal cells with α-1,6-fucosyl transferase (FUT8). Some mouse cell lines are characterized by a lower level of this ferment than CHO. However, the most efficient method to eliminate fucose is inactivating the FUT8 gene. CHO lines with inactivated FUT8 genes allow the construction of non-fucosylated therapeutic antibodies with aCDC increased 50-200 times [67]. The explanation of the role of the IgG carbohydrate component gave the impetus to a new direction in antibody engineering. Today, technologies of antibody construction, taking into account the carbohydrate component, are making rapid progress [68]. This direction is considered the most promising for constructing next-generation therapeutic antibodies with improved characteristics. 

## 6. BISPECIFIC ANTIBODIES 

**Fig. 4.**
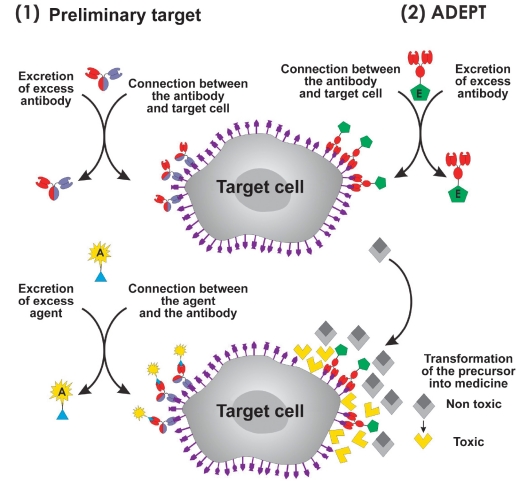
Scheme of two-stage target-killing technologies. (1) Pretargeting using antibodies with the following target killing by the involved a agent. (2) Preliminary delivery of E ferment to the target. at the second stage, E ferment transforms the nonactive medicine precursor into an active form (aDEPT, antibody-directed enzyme pro-drug therapy).

**Fig. 5.**
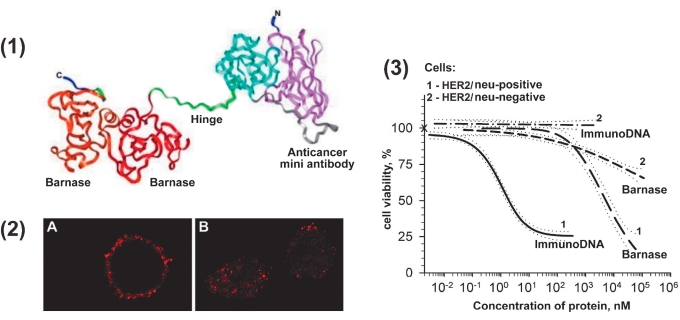
Immunodibarnase as a perspective agent for treating malignant neoplasms [105]. (1) Scheme of immunodibarnase structure. (2) Interaction of immunodibarnase with the cells expressing the HER2/neu cancer marker: surface binding at 4°C (a) and internalization at 37°C (B). (3) Cytotoxicity of immunodibarnase for the cells with hyperexpression of the HER2/neu cancer marker.

Natural antibodies are monospecific, i.e., they bind to antigens of only one species. antibody engineering technologies allow bispecific antibodies (Fig. 3, 2, 4, 5, 8) with a specificity to two different antigens to be created [29, 69]. The first bispecific antibodies were produced by fusing two hybridome cell lines. The quadroma created yields a random mixture of a target-oriented bispecific antibody and primary antibodies. Then, the required variant is obtained in the course of a complicated separation. a range of approaches was offered to increase the production of bispecific molecules, for instance, the creation of an interspecific mouse quadrome in the course of pairing the light and heavy chains of one species [70] and delicate "knob-and-holes" technology [32]. Two mutants were injected into the Mab CH3-domains: "the knob" for replacing the small amino-acid residue (threonine) with a bulky amino-acid residue (tyrosine) and "the hole," for reverse replacement. 

Fusing the hybridomes that produce two types of antibodies routinely led to the formation of bispecific molecules, which could be clicked-up by "knob-and-hole." Chemical conjugation [71] is still considered the most popular method of bispecific antibody creation, in spite of disadvantages such as the necessity of chemical modifications, which are occasionally harmful to antibodies, and the problem of separating the variants obtained. 

The gene engineering methods allow the creation of bispecific Mab and their fragments, avoiding the problem of separating the complex mixture produced [9]. The streptavidin-biotin system, characterized by stable association with a constant of KD 10^-15^ M dissociation, is traditionally used for these purposes. We offered a barnase-barstar protein pair (Fig. 3 4,5) as a heterodimerization module. This protein pair is characterized by a rather stable association with the KD 10^-14^ M dissociation, almost just like the streptavidin-biotin system, but makes it possible to produce bispecific antibodies with an exact ratio of 1 : 1 or 1 : 2 (trimer) and does not require any chemical modification of the components [72], which is essential for delicate proteins such as antibodies. Recently, a new sophisticated method called "dock and lock" (the DNL-method) was developed to create bispecific antibodies [33] (see also Section 7). antibodies produced by this method are distinguished by good pharmacokinetic characteristics and bivalent binding with surface antigen; thus they can be regarded as perspective agents for radioimmunotherapy. However, the major disadvantage of this method is the low affinity of the (KD ~ 10^-9^) complex, which requires additional stabilization by disulfide bonds. 

Bispecific antibodies were primarily used for retargeting the cytotoxic immune cells on the pathologic targets. Numerous bispecific antibodies were created: one identification domain of an antibody was bound to a characteristic surface marker on the cancer cell, while another domain was bound to an activation receptor of the cytotoxic cells [69, 73]. IgG FcyRI (CD64), characteristic of monocytes, macrophages, and dendritic cells; Iga FcαRI (CD89), expressed largely in neutrocyte, macrophages, and acidocytes; and the CD3 marker of cytotoxic T-lymphocytes were used as receptors which can attract cytotoxic cells to the target. It should be noted that T-lymphocyte, one of the most efficient killer cells, cannot be attracted to antibodies due to a lack of Fcy receptors. Unfortunately, clinical testing established the shortcomings of that method. Immune cells needed additional activation, an effect that appeared only at a 40- to 100-fold surplus of effector over the number of target cells and high dose of medicine, and, finally, high dose-limiting toxicity in medicines was observed [73, 74]. The use of bispecific antibodies for attracting cytotoxic T-cells to the cancer targets by binding with CD3 was effective only upon local application [75] and on the adaptive transfer of T-cells processed by bispecific antibodies ex vivo [76]. 

Almost all those obstacles were overcome by virtue of a new generation of bispecific T-cell engagers (BiTE), which represented recombinant bispecific single-chain antibodies composed of two scFv-fragments specific to the surface antigen of the target cell and T-cell CD3 antigen. In contrast to the above-mentioned full-size bispecific Mab, BiTE antibodies do not require the additional activation of attracted immune cells and have enough cytotoxicity at much less concentrations [77, 78]. Obviously, bispecific Mab, which retargets immune cells, can be a perspective direction especially for the treatment of cancerous blood diseases. Clinical investigations show that the application of BlinatumoMab (anti-CD19/anti-CD3) medicine in a concentration of 0.005 mg/m2/day leads to the complete elimination of cancer B-lymphocytes from the blood flow of non-Hodgkin lymphoma patients [79].

Analogous methods involving bispecific antibodies were used to retarget stem cells, viruses, and pathogens. Bispecific antibodies, which identify the receptor of the CD45 stem cells, and antigens, which become readily accessible in case of myocardial infarction [71], were suggested as a way to attract the stem cells to the myocard of an experimental animal suffering from a heart attack and for the regeneration of vascularization. This approach allows the delivery of a greater number of cells than injections of the stem cell suspension directly into the myocard. The researchers also tried to use bispecific antibodies for directed delivery to adenovirus tumors for gene therapy [80] and to eliminate pathogens from the blood flow, retargeting them on the CR1 receptor on erythrocytes [81]. These investigations are far from complete and will be subject to clinical trials. 

The second considerable sphere of clinical application of bispecific antibodies is pretargeting with the following directed delivery of toxins, chemotherapeutic agents, and radioactive isotopes to the cancer cells (Fig. 4). The idea of pretargeting with the following elimination "on a tip" is based on the desire to maximally decrease the global toxic effect when using strong toxins and radionuclides at the cost of both less circulation time in the blood and a decrease in the toxin therapeutic concentration. For this purpose, at the first stage, antibodies that are focused on a target - for instance, a tumor - are injected into an organism; then, after the natural removal of antibodies unbound to the target, the second cytotoxic component (toxin, radionuclide) is injected and specifically binds to the injected antibodies (Fig. 4). Initially, unmarked bispecific antibodies were suggested to be used for pretargeting: one valency would be bound to the tumor antigen, while another valency would be bound to the hapten carrying a radioactive isotope [82]. The disadvantage of this method is the monovalent non-optimal binding to the antigen, as well as the relatively low production of bispecific antibodies obtained from the myelome line cells. The gene-engineering methods made it possible to construct a range of truncated antibody fragments for radioimmunotherapy, including multivalent fragments (see Sections 1, 2, and 7), which led to a significant improvement in the pharmacokinetic properties of the targeting antibodies, specificity, and resolution of the method [83].

The third direction, which requires the construction of bispecific antibodies, is the simultaneous binding of two different antigenic determinants on one target. The higher density of the surface antigen on the target cells compared to the nonpathogenic cells is an essential condition for using this antigen for targeting; this provides specific action on the target and the safety of the whole organism. The number of molecules on the cell's surface is often insufficient. In this case, one way to increase the specificity of targeting on the cancer cell and the time of antibody retention on the target is to construct bispecific antibodies capable of simultaneous binding with two different tumor-associating antigens expressed on the same cancer cell [72, 84]. This method is important, because additional surface antigens can appear in the treatment process in response to the blocking of EGFR or HER2/neu receptors and cause resistance to medicines such as Trastuzumab, CetuxiMab, and Panitumumab. Recently, it was shown that resistance mechanisms may be activated due to a surplus of other tyrosine kinase receptors (IGF-RI, c-MET, Ron, etc.), which activate the same signaling pathways as EGFR and HER2/neu, bypassing the blocked receptors [85]. Using bispecific antibodies for the simultaneous blocking of EGFR and IGF-1R significantly intensified the response of cancer cells when compared with monospecific antibodies [12]. This combined action on several surface antigens can block surplus signaling pathways in the cancer cell and lead to a more successful treatment. a combined effect is also produced by bispecific antibodies such as "intrabodies," which are specific to the VEGF-R2 and Tie-2 angiogenesis receptors. The simultaneous blocking of two angiogenesis pathways by intrabodies intensifies the inhibition of angiogenesis and tumor growth more significantly than the blocking of only one pathway [86].

## 7. IMMUNOCONJUGATES 

The goal of achieving a "magic bullet" and the low efficiency of "unloaded" antibodies relative to the targets induced researchers to conjugate antibodies with other effector molecules (Fig. 3): radioisotopes, toxins, interleukins, and ferments activating medical products. In these immunoconjugates, antibodies usually act as a targeting component which delivers an active (cytotoxic) or diagnostic agent to the target. Chemical conjugation methods are commonly used for the conjugation of low-molecular agents, for instance, radioisotopes or low-molecular fluorescent dyes [11, 87]. Cell-penetrating peptides (CCP), capable of penetrating the membrane and transferring other proteins inside a cell, are conjugated to antibodies in the tumor by gene engineering in order to increase the penetration degree of radioimmunoconjugates. The most efficient cell-penetrating peptides are penetratine, which represents oligopeptide (43-58 a.r.) from the homeodomain of the drosophila antp protein, and TaT, which is an oligopeptide (49-57 a.r.) from the transactivator of HIV transcription. Mini-antibody radioimmunoconjugates, which are specific to the TaG72 tumor antigen and provided with penetratine or TaT oligopeptide, are accumulated 2.5-3 times better in the tumors of xenographic mice [88].

In oncology, two medical products are approved for the radioimmunotherapy of non-Hodgkin's lymphoma: Bexxar©, which is mouse IgG2a conjugated with β-emmiter of medium energy 131I (the penetration radius is 1 mm), and Zevalin©, which is mouse IgG1 conjugated with β-emmiter of high energy 90Y (the penetration radius is 11 mm) (Table 2). Currently, investigations are focused on replacing mouse antibodies as targeting agents for radioimmunotherapy with less immunogenic fragments (see Section 3), as well as on developing pretargeting technologies to decrease the general radiation load on the organism (see Section 6). 

To achieve these goals, the heterodimerization module is required, as with the creation of bispecific antibodies. Today the streptavidin-biotin system [89], which is well-proven for a range of analytical applications, is being used for this purpose. However, using this system in the human organism is limited by its high content of endogenic biotite, which can compete with the biotin-modified components. We have offered a new strategy of two-stage delivery based on ribonuclease, barnase, and its inhibitor, barstar [30, 31]. as was mentioned earlier that, these two proteins (110 and 89 a.r.) form the (KD ~ 10^-14^ M) complex, which is similar in resistance to the streptavidin-biotin system [90]. The proteins are stable; well-soluble; resistant to proteases; and, thus, compatible to the bacterial expression system. The N- and C-tails of both proteins occur outside the interaction area and are readily accessible for gene engineering fusion with the targeting mini-antibodies and cytotoxic agents [91, 92]. The principal possibility of the two-stage delivery of an active agent to the cancer cells was shown using the example of recombinant mini-antibodies, HER2/neu specific to the cancer surface antigen and conjugated with barstar, and a visualization component such as a recombinant fluorescent protein - EGFP - conjugated to barnase [31]. The essential advantage of the barnase-barstar module is the exact 1 : 1 ratio of components in the complex and the absence of self-aggregation, as well as the high affinity of interaction, which exceeds the values of all other dimerization systems (except for the streptavidin-biotin one). But, in contrast to the streptavidin-biotin system, the use of a heterodimerization module is based on gene engineering technologies and does not require any covalent modifications. 

The DNL method is of special interest among other methods developed for pretargeting a target cell [33]. This technology is based on the specific protein-protein interaction of a dimeric regulatory subunit (RII) of cyclic adenosine monophosphate-dependent protein kinase and the anchoring domain (amphipathic helix from 14-18 a.r.) of an a kinase anchor protein. a trivalent bispecific antibody focused on carcinoembryonic antigen (CEa, tumor antigen) and hapten histamine-succinyl-glycine was constructed on the basis of those polypeptides and the Fab-fragments of two different antibodies. at the first stage of directed delivery (Fig. 4), the "landing" component provides the bivalent interaction with the tumor antigen. at the second stage, the "lock," connected by the anchoring domain to the "dock," is bound to the hapten, which is marked by radioactive 99mTc. The relatively low affinity of the (KD ~ 10^-9^) complex requires additional stabilization by disulfide bonds. One more disadvantage is the complexity of the trivalent antibody construction composed of five different protein chains. In spite of these problems, pretargeting made it possible to increase the accumulation of the radioactive mark on the tumors grafted to mice by ~25 times when compared to the one-stage injection [33]. Today, this method can replace the bispecific Mab and streptavidin-biotin system commonly used for pretargeting [89]. Indeed, the trivalent bispecific antibodies created by the "dock and lock" method have a range of undeniable advantages for this application: bivalent binding with a tumor antigen, which intensifies the interaction and increases the time of retention on the pathogenic cells; the absence of the antibody effector functions, which significantly reduces unwanted side effects; the rapid removal of the targeting component from the organism, which decreases the waiting time for the second stage - the injection of the radioactive isotope proper - from 6-7 days to a few hours [93]. 

Much research is devoted to using different strong toxins for directed action on the target cells; 44% of medicines with anti-tumor antibodies subject to clinical trials are immunoconjugates or recombinant proteins. However, only one medicine, Mylotarg©, which represents a humanized anti- CD33-antibody GentuzuMab chemically conjugated with cytotoxic antibiotic calicheamicin, was approved for clinical application (Table 2). This disproportion reflects all the objective problems appearing in researchers' way when developing such an attractive idea. at the first stage of investigation, immunotoxins constructed on the basis of bacterial toxins or doxorubicin antibiotic were characterized by high systematic toxicity and a great number of side effects. Using antibodies loaded with strong toxins requires a more careful approach to selecting a target, because it is essential to avoid delivering cytotoxic agents to normal cells. It is preferable to target immunotoxins on antigens that are not expressed on normal cells but are readily expressed on the surface of tumorous cells. Unbound immunotoxin should be eliminated from the blood flow as soon as possible. The rapid internalization of antigen after binding with immunotoxin is preferable as well. Moreover, because a medicine's efficiency is related to the compound's toxicity level, it is expeditious to use toxins with a IC50 level which ranges within the nano- and picomolar concentrations. To date, researchers have the following range of required toxins: auristatin and calicheamicin, as well as such protein toxins as pseudomonade ricin and diphtherin [94]. 

All these ideas were taken into consideration in designing Mylotarg© (Table 2). Humanized Mab GentuzuMab is specific to surface CD3 antigen, which is hyperexpressed on the surface of malignant cells caused by acute myeloid leukemia; therefore, a maximum non-toxic dose of this medicine is rather high and attains 9 mg/m2. CD33 is readily internalized, and the therapeutic response depends not on the antigen expression level but on the stage of the cell cycle and multi-drug resistance. The cytotoxic component of this immunotoxin is the antibiotic calicheamicin, which binds to the DNa groove and breaks it. The toxicity of this antibiotic is characterized by the IC50 value, which is within the low nM concentration.

The cytotoxicity of antibiotics from the auristatin and maytansinoid group has another nature. These antibiotics bind to α-tubulin and break the polymerization of microtubules, which causes cell-cycle block and cell death. CD20, CD30, CD70, PSMa (prostate-specific membrane antigen), HER2/neu, E-selectin, and LewisY cell markers are used as the targets for immunoconjugates. as in the case with radioimmunotherapy, immunoconjugates are more efficient for treating hematologic types of cancer and are inefficient for the treatment of solid tumors. 

Currently, immunotoxins derived from truncated pseudomonade a exotoxin and diphtherin are subject to clinical testing. The action mechanism of these immunotoxins is based on the catalytic aDP-ribosilation of factor 2, inhibiting the translation. The first and the second generations of those multidomain proteins were characterized by high systematic toxicity, which significantly decreased after the elimination of the domains responsible for binding with the cells. The major advantage of these proteins is their high toxicity in the pM concentrations: only a few molecules are needed to kill a cell. The disadvantage is the immunogenicity and presence of side effects such as the capillary leak syndrome. To inhibit immunogenicity, A. Pastan, a pioneer researcher in immunotoxins, carried out hard work focused on the deimmunization of truncated pseudomonade PE38 toxin with the complete retention of its toxicity [95]. The high intensity of his investigations aimed at creating medicines on the basis of immunotoxins will likely make them available for clinical use very soon. 

Attempts to use RNaase as toxins were made much later, at the end of the 1990s. The researchers obtained conjugates of bull pancreatic RNaase and anti-EGFR mouse antibodies [96], as well as a recombinant protein on the basis of human pancreatic RNaase and single chain anti-transferrin receptor mini-antibodies [97]. Human pancreatic RNaase was conjugated with a humanized mini-antibody to decrease immunogenicity [98]. The same authors were the first to offer the term "ImmunoRNaase." Today, immunoRNaase is of great interest [99-102]. RNaase draws the attention of researchers due to its accessibility, low immunogenicity, and absence of toxicity outside the cell. Thus, it is quite possible that RNaase-based medicines will not be characterized by systematic toxicity, but the problem of internalization into the target cells remains unsolved. Using human RNaase is significantly limited by inhibition by a natural inhibitor (RI) present in the cells. Researchers produced mutants of human RNaase resistant to the inhibitor [103] by directed mutagenesis and offered a search for the appropriate RNaase of other origins [104]. Currently, the clinical testing of onconase, the first RNaase used for cancer therapy [105], is drawing towards a successful completion. This positively charged protein (104 a.r) from frogs belongs to the amphibian a ribonuclease superfamily and has cytostatic and cytotoxic action largely on cancer cells. This selectiveness is likely related to the strong positive charge of onconase and the fast metabolism of the cancer cells [105].

In our laboratory we used the bacterial ribonuclease of barnase to create immunotoxin [30, 45, 106, 107]. This small, stable, and well-soluble protein is resistant to the natural inhibitor of human RNaase. Earlier, we established that the barnase of multidomain recombinant proteins plays the role of internal chaperone, provides the correct domain coiling, and favors the stability and good solubility of protein [108]. Barnase, which is toxic for cells, has been used to create a zero-background vector [90]. The constructed immunodibarnase contains two barnase molecules and a humanized mini-antibody specific to the internalized cancer HER2/neu marker (Fig. 5, 1). The in vitro experiments show that providing a toxic barnase agent that addresses the mini-antibody significantly increases the efficiency of action on the cancer cells. The anti-cancer mini-antibody delivers barnase on the surface of cancer cells (Fig. 5, 2(a)) and provides for its penetration into the cell (Fig. 5, 2(B)), where it exposes its toxic nature. Immunodibarnase (1.8 nM) causes the apoptosis of 50% of HER2/neu-positive cells, whereas HER2/neu-negative cells require a concentration 250-300 times higher (Fig. 5, 3). The cytotoxic effect of barnase depleted in mini-antibodies is non-specific and ~1000 times less acute than the cytotoxic effect of immunodibarnase (Fig. 5, 3) [107]. Immunodibarnase can be a perspective agent for treating tumors thanks to its specific action on the cancer cell and low active concentration. 

RNAase circulating in the blood stream is nontoxic to the organism and becomes toxic only after penetrating into the cancer cell, where it plays the role of a so-called protoxin [99]. a similar idea would be to create nonactive pre-medicine which would be delivered to the target and then transformed into active form; this is being developed in different variants on the basis of aDEPT technology (antibody-directed enzyme pro-drug therapy) (Fig. 4). The technology is based on the preliminary directed delivery of a ferment conjugated with the antibody to the target cells. Targeting the delivery is provided by the especificity of antibody to the cell antigen. at the next stage, in the microenvironment of the target, the injected non-active precursor of the medicine is transformed into an active form by a ferment delivered earlier and kills the surrounding cells. β-lactamase, G2 carboxypeptidase, and others are used as ferments at this stage [86, 109, 110].

Creating anti-idiotypic antibodies with definite catalytic functions-so-called abzymes-which can perform the functions of antibodies and are characterized by fermentation activity is a new prospective scientific direction of gene engineering. Modifying the anti-idiotypic antibody scFv-fragments, combined with the use of phage libraries, makes it possible to select abzymes with preprogrammed properties [111, 112]. 

Some cytotoxins (for instance, IL-2, IL-12, TNFα, IFNγ, GM-CSF) have immunomodulatory and antitumoral value. Unfortunately, using these proteins as medicines is hardly possible due to their high systemic toxicity in active concentrations, fast decomposition and removal from the organism, and the undirected and nonspecific action on the tumor cells. The conjugation of antibodies with cytotoxins and the creation of immunocytotoxins makes it possible to overcome the limits pointed out. Investigating interleukin-2 therapeutic and adjuvant properties brought about the greatest step forward. For instance, we have obtained encouraging results in the course of clinical trials of recombinant immunocytokines constructed on the basis of interleukin-2, anti-cancer humanized antibodies (GD2 disialoganglizid and the epithelial cell adhesion molecule EpCaM), and anti-angiogenesis antibodies (EDB and the extra domain B of fibronectin) for treating melanoma, neuroblastoma, and non-Hodgkin's lymphoma [113, 114] and on the basis of interleukin-2 conjugated with anti-CD20 antibody for treating B-cell malignancy [115]. Using immunocytokines helps increase the effectiveness of chemotherapy as well [116]. 

Conjugates of antibodies with different fluorescent probes are often used in immunology for in vitro investigations. Introducing a powerful instrument such as fluorescent proteins by molecular biology (2008 Nobel Prize, Dr. Osamu Shimomura, Dr. Martin Chalfie, and Dr. Roger Y. Tsien) allows us to observe the vital activity of the cells on a real-time basis. Japanese researchers created a system for visualizing living cancer cells in the organism based on the pH-activation fluorescent agent directed to the target cell by an anti-cancer internalized antibody [117]. Fluorescence is observed only if the agent occurs in the lysosomes of the living cancer cell and disappears when it dies. The method's specificity is 99%. These investigations are far from being used in therapy, but they enable us to discover the new abilities of immunoglobulin conjugates. 

## 8. RECOMBINANT ANTIBODY CONSTRUCTION SYSTEM.

Today, producing stable and high-affinity Mab in a sufficient amount for pre-clinical and clinical investigations is a "bottleneck" for the wider application of these therapeutic compounds. The rapid growth in demand for antibodies and their quality has led to the development of numerous systems for producing antibodies and their fragments on the basis of Gram-positive (Bacillus) and Gram-negative (Escherichia) bacteria, ferments, filamentous fungi, and the cell lines of insects and mammals [118-120]. High-technological systems such as bacterial and ferment producers, which favor the overgrowth of biomass in fermenters and the production of highly efficient recombinant proteins, can solve the problem of creating truncated Mab fragments, non-glycosylated Fab-fragments, and scFv provided, as a rule, with special tail peptides for fast activation on affine sorbent. Currently, antibodies are created in transgenic plants and animals which are appropriate for the production of full-size antibodies [72, 121]. The expression system is created on the surface of bacterial cells (so called "E-clonal" antibodies) used for both full-size and divalent formats [122] for the fast production and selection of full-size antibodies. The glycosylation of antibodies in ferments, plants, and insects differs from that in a human being: therefore, antibodies obtained in those systems are applicable only for experimental investigations. Today, therapeutic antibodies are produced in transgenic mice, whose immunoglobulic locuses are inactivated and replaced with the genes of human immunoglobulins [10, 123]. The most well-known mice transgenic lines are as follows: Xenomouse (abgenix, www.abgenix.com), HuMab mouse (GenPharm, www.genpharm.ca), and TC mouse and KM mouse (Kirin Brewery Company, www.medarex.com). Medicines with Mab created in these producers are characterized by low immunogenicity due to xenogenic post-translation modifications and, primarily, to glycosylation specifics. Moreover, the researchers are developing new technologies which would allow the production of Mab with a human profile of glycosylation in ferments, insects, and transgenic plants [124, 125]. 

## 9. CONCLUSIONS AND PROSPECTS.

In the early 21st century, one hundred years after Ilya Mechnikov and Paul Ehrlich, the founders of the current immunity theory (1908), received the Nobel Prize, knowledge about the delicate molecular mechanisms of antibody functioning and interaction with the organism's protective systems has made the greatest step forward. This progress is provided by the innovative technologies used in the course of scientific investigations and the accumulation and systematization of large amounts of information. The quick development of bioinformatics allows us to model the compounds with preprogrammed properties, while revolutionary progress in genetic and cell engineering technologies makes it possible to create biotechnological producers of therapeutic medicines. 

Modern technologies make it possible to modify the antibody properties depending on the situation and use all their functions. Thanks to gene engineering, researchers manage to decompose antibodies and compose new constructions. Truncated antibodies, focused on binding with a target and compatible with a bacterial expression, are used for producing a wide range of compounds meant for the highly efficient delivery of active agents. Researchers have created clone libraries of immunoglobulin fragments and highly efficient screening systems. Moreover, a new scientific direction has appeared; it is focused on constructing framework molecules, also called scaffolds (as an alternative to antibodies), which are characterized by an analogous ability to specifically bind. Undoubtedly, this sphere will develop in both directions: the construction of the next generation of recombinant truncated humanized antibodies with multivalent binding characterized by improved pharmacokinetic properties [34, 126] and the creation of alternative framework constructions [127]. Truncated antibodies that are depleted in constant domains are widely used in radioimmunotherapy for the directed delivery of radioactive isotopes as immunotoxin components in oncology and as specific blockers of targets in cardiosurgery. 

The bispecific antibodies in both truncated and full-sized formats have great potential. These antibodies have been used in radioimmunotherapy for a rather long time, and new technologies can only modify and improve the currently available and well-established methods of one- and two-stage delivery of radioisotopes to a target [93]. On the contrary, using bispecific antibodies in the sphere of cell immunotherapy has just begun [128], and researchers have a wide field of experimental and clinical investigations. The high variability of the cancer cells and the problem of resistance to therapy generate a need for complex treatments and the use of several medicines with different mechanisms of action. Bispecific antibodies, which can strike two targets simultaneously and block two metabolic pathways, are irreplaceable in this case as well. 

New technologies for controlling the functions of the antibody constant domain are reaching new heights. The development of constant domain engineering and, in particular, carbohydrate Mab engineering testifies to a return to "traditions" and growth in interest in the antibody effector functions and full-size Mab, because they attract all the protection systems of the organism to the pathological center [129]. 

One scientific direction which is quite promising from the technological standpoint is the creation of hybrid biocompatible nanoparticles from organic and inorganic material. Providing these nanoparticles with antibody particles will ensure high-accuracy targeting. Semiconductive fluorescent crystals ("quantum dots"), magnetic nanoparticles, gold colloids, and fullerene derivative delivered with their help will carry medicinal compounds and allow additional external action on the targets by means of laser, acoustic, and microwave radiation [130, 131]. This new generation of multifunctional nanoconstructions should have complex properties that cannot be used separately. This combined action on the tumors will help achieve a whole that is truly more than the sum of its parts. 

Today, only 15 out of 266 applied medical targets found in the human genome are used as antigens in making therapeutic antibodies to treat different diseases, and almost all of them are surface cell antigens [132]. accumulating knowledge about cancer biology will make it possible to reveal new cell chains and directionally create multifunctional constructions for accurate action against the targets responsible for the profileration, conglutination, metabolism, distribution, and other mechanisms of malignant neoplasms (see, for instance, [133]). 

It can be expected that the future success of the clinical application of antibodies will depend to a great extent on the development of new targets for them in the course of accumulating new knowledge about the mechanisms and molecular participants of the pathological processes.

## Acknowledgements

S.M. Deyev's work at the antibody Engineering Laboratory was supported by the Russian Foundation for Basic Research (project no. 09-04-01201-a), the Federal Target Program for the Research and Development of Priority Directions of Scientific and Technological Complex in Russia in 2007-2012, and the "Molecular and Cell Biology" and "Fundamental Research of Nanotechnologies and Nanomaterials" programs of the Russian academy of Sciences Presidium.
